# Supplementary material for: Hepatic Failure in COVID-19: Is Iron Overload the Dangerous Trigger?
Source: Cells. 2021 May 4;10(5):1103. doi: 10.3390/cells10051103 (PMC8147922; doi:10.3390/cells10051103)
Supplement: Supplementary file 1 [file cells-10-01103-s001.zip › cells-1132430-supplementary.pdf]

Supplementary Figure 1

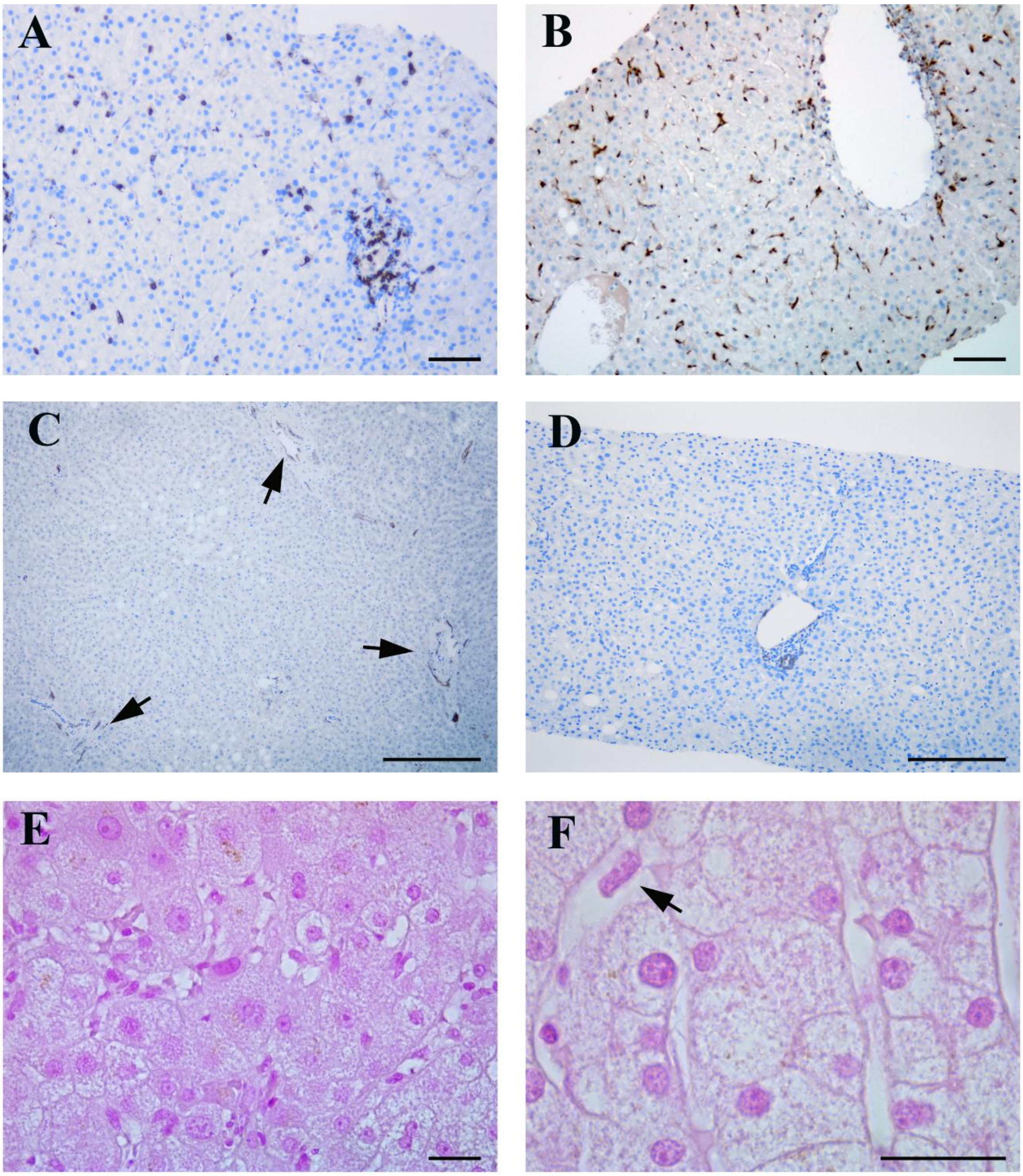

Supplementary Figure 1

Representative photomicrographs of immunohistochemical and Perl's staining in control liver. Scattered CD8+ lymphocytes (A) and CD68+ macrophages (B) are present; (C) CD34-positive staining was observed in vessels of portal area, while CD34 labelling results negative in normal liver sinusoids; (D) αSMA-positive staining was limited to the vascular walls in the portal areas (arrows).

(E) The absence of blue granules after Perl's staining revealed the absence of iron deposits, neither in hepatocytes (H) nor in Kupffer cells (arrow) (D).

Scale bars: A,B=14 μm; C,= 100 μm; D=50μm; E,F= 7 μm.

Supplementary Figure 2

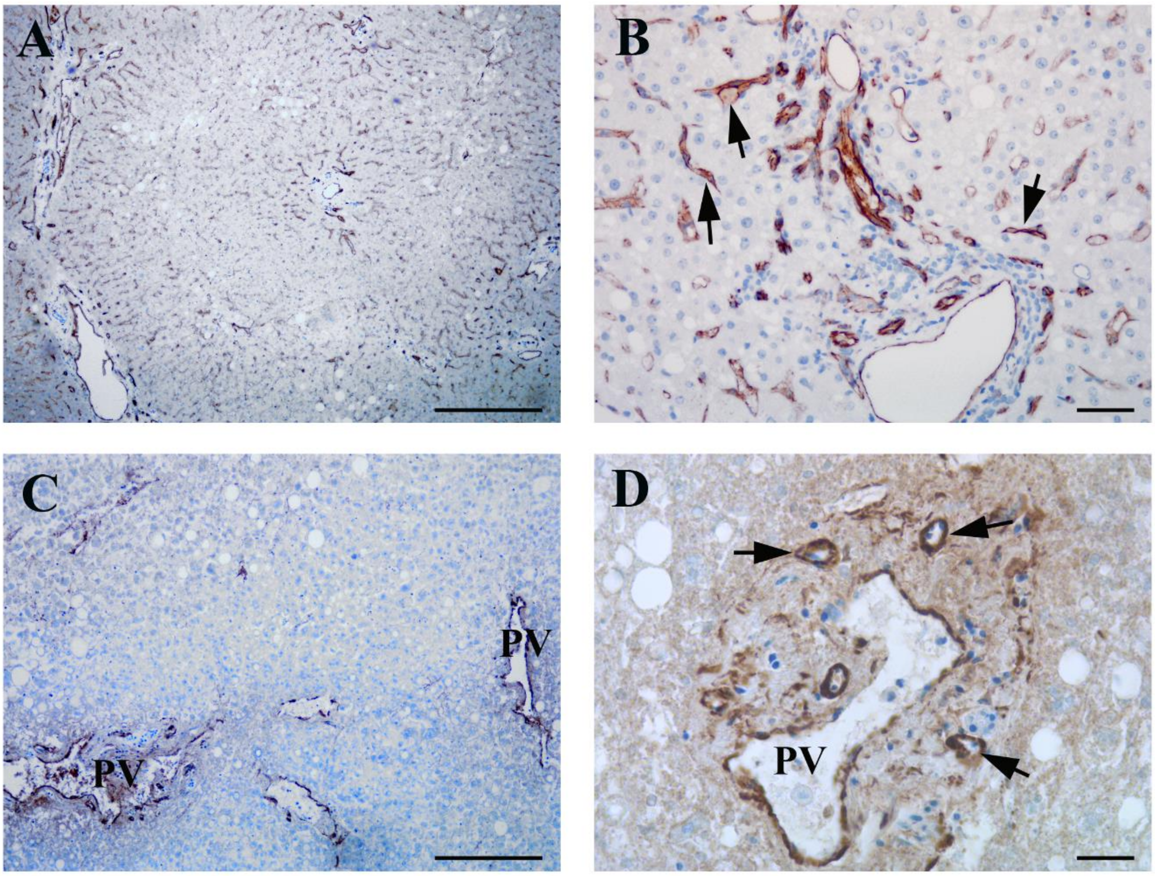

Supplementary Figure 2
Expression of von Willebrand factor (vWF) and alpha-smooth muscle actin (αSMA) in liver sections from COVID-19 patients.
(A,B) vWF positivity is visible all along portal vessels and centrilobular vein; positive stain is also present in dilated sinusoids (arrows).
(C,D) αSMA strong positive staining of portal vein (PV) walls and of aberrant vessels branches in the portal tract (arrows). Scale bars: A=100 µm; B,D,= 14 µm; C=50µm.
